# Acid‐Resistance and Self‐Repairing Supramolecular Nanoparticle Membranes via Hydrogen‐Bonding for Sustainable Molecules Separation

**DOI:** 10.1002/advs.202102594

**Published:** 2021-10-19

**Authors:** Wang Han, Ming‐Jie Yin, Wen‐Hai Zhang, Zhi‐Jie Liu, Naixin Wang, Ken Tye Yong, Quan‐Fu An

**Affiliations:** ^1^ Beijing Key Laboratory for Green Catalysis and Separation Department of Environmental and Chemical Engineering Beijing University of Technology Beijing 100124 China; ^2^ The University of Sydney Nano Institute The University of Sydney Sydney New South Wales 2006 Australia; ^3^ School of Biomedical Engineering The University of Sydney Sydney New South Wales 2006 Australia

**Keywords:** acid‐resistance membrane, hydrogen bond, molecule separation, self‐healing materials, supramolecular nanoparticle materials

## Abstract

Functional membranes generally wear out when applying in harsh conditions such as a strong acidic environment. In this work, high acid‐resistance, long‐lasting, and low‐cost functional membranes are prepared from engineered hydrogen‐bonding and pH‐responsive supramolecular nanoparticle materials. As a proof of concept, the prepared membranes for dehydration of alcohols are utilized. The synthesized membranes have achieved a separation factor of 3000 when changing the feed solution pH from 7 to 1. No previous reports have demonstrated such unprecedentedly high‐record separation performance (pervaporation separation index is around 1.1 × 10^7^ g m^−2^ h^−1^). More importantly, the engineered smart membrane possesses fast self‐repairing ability (48 h) that is inherited from the dynamic hydrogen bonds between the hydroxyl groups of polyacrylic acid and carbonyl groups of polyvinylpyrrolidone. To this end, the designed supramolecular materials offer the membrane community a new material type for preparing high acid resistance and long‐lasting membranes for harsh environmental cleaning applications.

## Graphical abstract

Size‐tunable hydrogen‐bond supramolecular nanoparticles are synthesized for separation membrane fabrication, which can work stably at pH = 1 condition and exhibit self‐repairing ability.

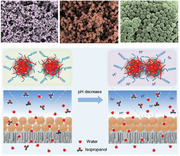

## 1 Introduction

Membranes have been broadly applied in the fields of gas separation, nanofiltration, ultrafiltration, as well as pervaporation,^[^ ^1^, ^2^, ^3^, ^4^, ^5^, ^6^ ^]^ which could reduce 90% energy consumption if replacing traditional distillation technique.^[^ ^7^, ^8^ ^]^ Nevertheless, the harsh components in the wastes, e.g., strong acids, will inevitably degrade the performance or even shorten the lifespan of membranes during long‐term running, hampering their practical applications.^[^ ^9^, ^10^, ^11^ ^]^ For example, the microstructure of industrially used zeolite NaA membranes will be destroyed at acidic condition (pH < 6), losing the separation ability with feed solution pH = 2.^[^ ^12^, ^13^ ^]^ As a result, lots of membranes can merely be used at neutral conditions in industry, though numerous applications emergently asking for acid‐resistance membranes, e.g., acidic mixed gas separation,^[^ ^14^ ^]^ bioethanol mixtures,^[^ ^15^ ^]^ biological membranes,^[^ ^16^ ^]^ pervaporation coupled esterification reactions, and so on. However, the physical or chemical bonds will be broken at strong acid conditions, it is still a bottleneck issue to develop advanced functional membranes that can function normally or even better at low acidic environment.

Aiming at promoting the application of membranes at acidic conditions, several strategies have been employed to fabricate acid‐resistance membranes, including tailor‐made chemical structures,^[^ ^17^ ^]^ crosslinking,^[^ ^18^ ^]^ surface modification,^[^ ^19^ ^]^ and developing novel materials.^[^ ^10^ ^]^ Crosslinking has been well‐recognized as a facile and efficient approach due to its potential large‐scale manufacture ability.^[^ ^20^, ^21^, ^22^ ^]^ For instance, polyelectrolyte complex membrane via electrostatic crosslinking was adopted for dehydration of alcohols at acidic conditions, which significantly facilitates the pervaporation application of the membrane in biological fermentation process.^[^ ^23^, ^24^ ^]^ However, the separation performance of those membranes degrades at low pH, e.g., pH < 3, with running time due to the weakened complexation strength at acidic conditions.^[^ ^11^ ^]^ Recently, Zhao et al. developed PILs complexed membrane for acidic dehydration, which can work at pH = 1 condition, but the performance degrades during long time tests.^[^ ^10^ ^]^ Moreover, the membranes have to be unloaded and disposed if being fractured during operation process, polluting the purified solvents and halting the running of the whole setup.^[^ ^10^, ^11^, ^13^ ^]^

Self‐healing, a natural function in biology, has inspired and boosted a large number of studies on exploiting artificial materials with self‐repairing ability, which can further advance the materials application and potentially solve the above issue.^[^ ^25^, ^26^, ^27^, ^28^, ^29^, ^30^, ^31^, ^32^, ^33^ ^]^ Commonly, the self‐repairing function of materials are realized by strong noncovalent interactions including electrostatic interactions,^[^ ^31^ ^]^ dipole–dipole interactions,^[^ ^32^ ^]^ host–guest interactions,^[^ ^34^ ^]^ metal coordination,^[^ ^35^ ^]^ *π*–*π* stacking interactions,^[^ ^33^ ^]^ and hydrogen bond interactions.^[^ ^36^ ^]^ Taking advantages of the self‐repairing materials, several kinds of separation membranes have been devised, e.g., nanofiltration membrane, gas separation membrane, as well as pervaporation membrane.^[^ ^37^, ^38^, ^39^ ^]^ Albeit, those membranes cannot work under strong acidic conditions, e.g., pH < 3. Toward this end, it is of paramount importance to develop advanced membrane materials endowed with both self‐repairing and acid‐resistance features.

Polyacrylic acid (PAA), a pH responsive polymer, ^[^ ^40^, ^41^ ^]^ can be complexed with polyvinylpyrrolidone (PVP) via hydrogen bond and formed supramolecular materials which can accomplish self‐repairing process by immersing the materials in water.^[^ ^42^ ^]^ Thus, the PAA/PVP supramolecular materials could bear the function of acid‐resistance and self‐repairing, due to the reversible hydrogen bonds and acid‐induced complexation.^[^ ^42^, ^43^ ^]^ Nevertheless, the complexed bulk materials cannot be adopted for functional membrane fabrication at mild conditions because of their insolvable feature, hindering their advanced applications. In this study, we realized preparation of PAA/PVP complexed nanoparticles, by precisely manipulating the complexation condition of PAA and PVP,^[^ ^43^, ^44^ ^]^ which can be well dispersed in water. Taking advantage of this trait, multifunctional PAA/PVP supramolecular film with controllable thickness can be facilely obtained via vacuum filtration technique for pervaporation application. In strong contrast to previous reports,^[^ ^10^, ^11^, ^13^, ^17^ ^]^ the alcohol dehydration performance of PAA/PVP membrane was enhanced with pH reduction, and the membrane can be stably running for more than 100 h at pH = 1 feed solution. Besides, the damaged PAA/PVP membrane can recover their original performance after self‐repairing. Thus, the proposed membrane materials could remarkably extend the lifetime of polymer membrane and reduce the waste of materials, prominently saving the energy and cost industrially and promoting the sustainable development of society.

## 2 Results and Discussion

**Figure 1:**
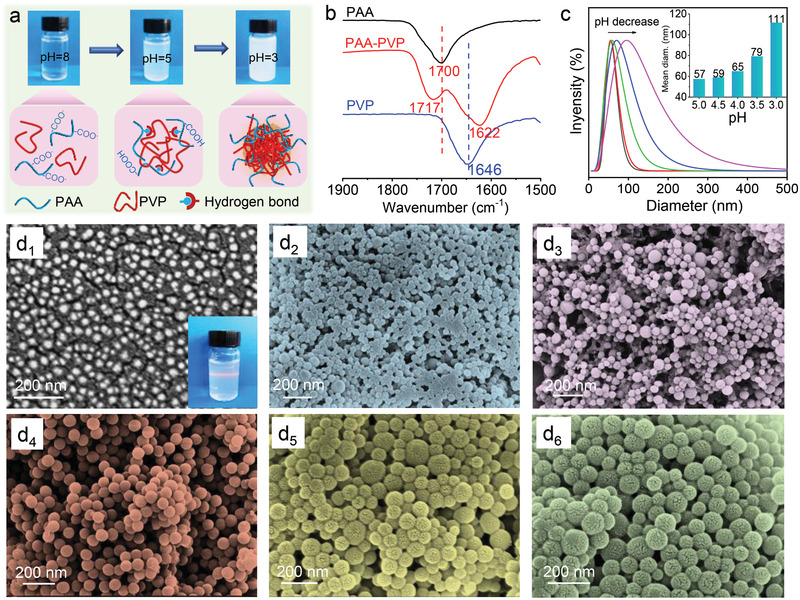
a) HCPN formation and nanoparticle size tuning via controlling the complexation pH. b) FTIR spectra of PAA, PVP, and PAA/PVP complex. c) The size evolution of swelled HCPN with different complexation pH. d) SEM images of the HCPN complexed at pH = 4 and dried at 40 ℃ for 2 h in the oven (d_1_, the inset shows the Dindar effect of the dispersed solution) and the change of swelled HCPN size with complexation pH (d_2_–d_6_).

**Figure** **1**a illustrates the hydrogen‐bonding complexation process of PAA and PVP: the two kinds of polymers can be well and uniformly dissolved in weak alkaline water (clean aqueous solution) and hydrogen‐bonding complexation between —OH of PAA and —C═O of PVP occurs with pH decline (white turbid solution). The complexed polymer chains form core–shell nanoparticles where the core is originated from highly aggregated PVP while the shell is the highly complexed PAA and PVP.^[^ ^42^ ^]^ The successful complexation between PAA and PVP is supported by the Fourier‐transform infrared (FTIR) in Figure 1b: the typical peak of carboxylic acid groups at 1700 cm^−1^ for PAA and the stretching vibration of the carbonyl groups peaking at 1646 cm^−1^ for PVP shift to 1717 and 1622 cm^−1^, respectively, after complexation.^[^ ^45^ ^]^

The scanning electron microscopy (SEM) image of hydrogen‐bonding complexed PAA/PVP nanoparticles (HCPN) formed at pH = 4 is shown in Figure 1d_1_: the size of the dried HCPN is around 40 nm. Notably, the size of the swelled HCPN can be widely tuned by controlling the complexation pH condition, where the size of swelled^[^ ^46^ ^]^ HCPN grows from 57 to 111 nm when pH declining from 5 to 3 (Figure 1c). The size of swelled HCPN change with environment pH can also be directly observed by SEM in Figure 1d_2_–d_6_, which shows clear particle size increase with pH reduction, in good agreement with the measured value by dynamic light scattering (DLS). The tunability of the HCPN is due to the pH dependent feature of PAA.^[^ ^40^, ^41^ ^]^ The carboxyl group of PAA will be protonated with addition of protons (pH decrease), facilitating the hydrogen‐bonding between PAA and PVP.^[^ ^20^ ^]^ As a result, the size of HCPN increases with more complexed polymer chains. This is corroborated by the FTIR (Figure S1, Supporting Information): both the characteristic peaks of carboxylic acid groups and carbonyl groups become broaden with pH decrease, hinting higher hydrogen‐bonding complexation degree.

With the eminently feature of acid‐induced complexation, the HCPN can be employed for fabrication of acid‐resistance polymer membranes via vacuum‐assisted filtration (Figure S2, Supporting Information). Here, dehydration of alcohols at acidic condition was chosen as a model application for HCPN membranes due to the emerging industrial requirement of highly acid‐resistance membrane. The HCPN was uniformly dispersed at aqueous solution, and filtrated on a commercial porous membrane (Figure S2, Supporting Information). After optimizing the membrane preparation conditions, e.g., complexation pH (Figure S3a, Supporting Information) and membrane thickness (controlling filtration volume in Figure S3b, Supporting Information), a complexation pH of 4 with membrane thickness of 430 nm (Figure S4, Supporting Information) was adopted for the subsequent study.

**Figure 2:**
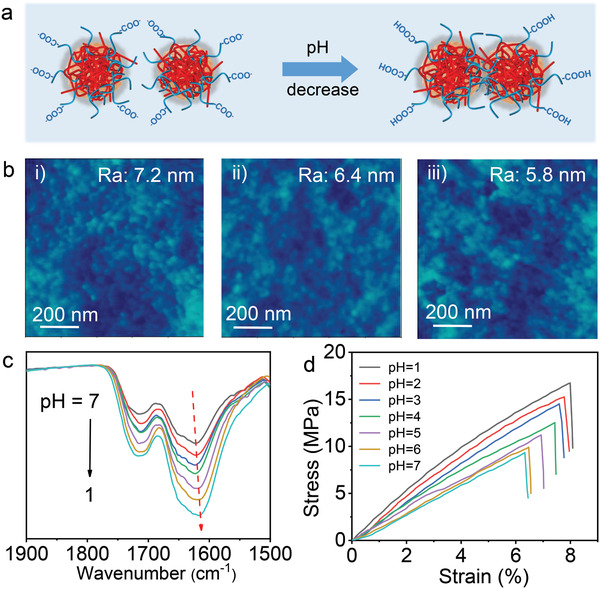
a) Scheme for the hydrogen‐bond crosslinking degree change among HCPN with pH reduction, which will enhance the acid‐resistance ability of HCPN membrane. b) Surface morphologies of the HCPN membrane measured by AFM after swelling at feed solution (10% water/isopropanol) with different pH: i) pH = 7; ii) pH = 4; and iii) pH = 1. c) FTIR spectra and d) mechanical properties variation of HCPN membrane treated with feed solution pH ranging from 1 to 7.

The surface of HCPN membrane is very smooth (root mean square (RMS) surface roughness = 2.6 nm, **Figure** **2**a and Figure S5b, Supporting Information) due to the small size of HCPN at dried state.^[^ ^10^ ^]^ Notably, obvious nanoparticle shapes can be observed after immersing the membrane with isopropanol/water feed solution, because of the confined swelling of HCPN,^[^ ^47^ ^]^ in agreement with the particle size increase at swelling state in Figure 1c,d. Interestingly, the surface morphology of the swelled HCPN membrane is closely dependent on the pH of feed solution (Figure 2b): the nanoparticles seem to melt together with pH declining from 7 to 1, meanwhile, the surface roughness of HCPN membrane decreases from 7.2 to 5.8 nm. The result indicates the hydrogen bond crosslinking degree becomes higher under stronger acidic condition. The increase of hydrogen bond intensity is also corroborated by the FTIR spectra variation and mechanical change of the HCPN with pH reduction: the peaks of the former become broader and shift a bit with pH decrease (Figure 2e) while the Young's modulus become larger with pH reduction (Figure 2f), which is promising for molecules separation under strong acidic condition.

**Figure 3:**
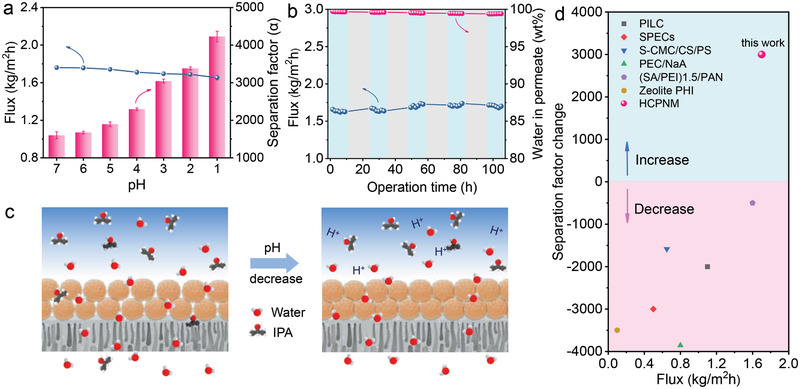
a) The performance of HCPN membrane in dehydration of 10% water/isopropanol mixtures with different pH at 50 ℃. b) Long‐term running stability of HCPN membrane in dehydration of 10 wt% water/isopropanol mixtures at 50 ℃. c) The scheme for the performance enhancement mechanism of HCPN membrane in acidic molecule separation. d) Performance comparison of HCPN membrane with other membranes for acidic pervaporation.

The separation performance of HCPN membrane was evaluated by dehydration of 10% water/isopropanol mixtures with different pH at 50 ℃. It should be emphasized that, in sharp contrast to previous studies, the selectivity of HCPN membrane gradually aggrandizes with lowering feed pH (separation factor increasing from 1500 to 4500 with pH reduction from 7 to 1, threefold enhancement, **Figure** **3**a), highlighting the salient acid‐resistance attribute of HCPN membrane (the HCPN membrane can work at alkaline conditions with reduced separation). Meanwhile, the flux decreases a little, from 1.76 to 1.65 kg m^−2^ h^−1^, but it is still among the top tier performance (> 1.50 kg m^−2^ h^−1^) for polymer membranes.^[^ ^10^, ^11^, ^13^, ^17^ ^]^ More importantly, it is the first report that the separation factor of polymer membrane in dehydration of alcohols raises with acid, reaching the unprecedentedly high separation factor at pH = 1 (Figure 3d and Table S1, Supporting Information).

The mechanism of the distinctively enhanced separation performance at acidic condition was investigated by the novel low‐field magnetic resonance (LFNMR) technique which is a well‐approved tool for examining the crosslinking degree (Figure S6a, Supporting Information).^[^ ^47^, ^48^ ^]^ Two peaks in the time‐domain of 0.1–30 ms can be obtained for HCPN membrane irrespective of the pH used for complexation (the shorter relaxation time means larger restriction to chain mobility): the first one is attributed to the hydrogen bond and the second one arising from noncomplexed polymer chains. Clearly, both of the two peaks shift to the shorter relaxation time, hinting the increase of hydrogen bond strength. In this regard, the free volume of HCPN membrane decreases, further hindering the passing through of isopropanol. Furthermore, the glass transition temperature of HCPN after treated with feed solution at different pH is enhanced 10 ℃ with pH decreasing from 7 to 1, as shown in Figure S6b, Supporting Information. The result further confirms the increase of complexation strength between PAA and PVP. Consequently, the separation factor was noticeably enlarged with flux declining a bit. The neglectable flux decline can be attributed to the partially water‐transportation blockage through the membrane, as confirmed by the water contact angle increase (Figure S7a, Supporting Information) and swelling degree decrease (Figure S7b, Supporting Information) of HCPN under acidic feed solution.

According to the above analysis, we propose the working mechanism of HCPN membrane under acidic feed solution. As illustrated in Figure 3c, at neutral conditions, part of PAA and PVP polymer chains in HCPN membrane are not crosslinked by hydrogen bond, because of the deprotonation of PAA. When the pH decreases, PAA chains will be protonated, providing active hydrogen atoms for hydrogen bonding with PVP.^[^ ^49^ ^]^ Thus, the crosslinking degree of HCPN membrane increases (the LFNMR data and differential scanning calorimetry (DSC) characterization in Figure S6, Supporting Information), hindering chains mobility and thus reducing the flexibility and free volume of HCPN membrane. Resultantly, both the isopropanol and water transportation are restricted, but the hinderance effect on water is not so obvious due to its small diameter,^[^ ^10^ ^]^ which accordingly amplifies the separation factor.

The prepared HCPN membrane can also work well at different separation conditions with a fixed feed pH = 1 to meet the practical application requirements, including feed solution concentration (Figure S8a, Supporting Information) and testing temperature (Figure S8b, Supporting Information). In particular, both the separation factor and flux are boosted with temperature. The pervaporation separation index (PSI) of HCPN in dehydration of 10% water/isopropanol mixtures (pH = 1) at 70℃ is 1.1 × 10^7^ g m^−2^ h^−1^, accomplishing a new record.^[^ ^50^ ^]^ Besides, the HCPN membrane can be utilized to dehydration of different azeotropic mixtures with different solubility in the membrane and molecule sizes, e.g., methanol/H_2_O, ethanol/H_2_O, as well as ethyl‐acetate/H_2_O (Figure S9, Supporting Information). Furthermore, the separation performance of HCPN membrane was also tested at pH = 1 condition continuously for more than 100 h (Figure 3b), no noticeable performance degradation can be observed in the whole process. Remarkably, as shown in Figure 3d, when changing the pervaporation condition from neutral to acidic solutions, the separation factor of our HCPN membrane was significantly enhanced around 3000. This is the first report that the separation factor increase with pH reduction while severely performance degradation occurred in previous reports.^[^ ^10^, ^11^, ^13^, ^17^, ^51^ ^]^ Considering the superior separation performance and long‐term stability of HCPN at rigorous conditions, the developed membrane remarkably accelerates the practical applications of polymer membranes.

**Figure 4:**
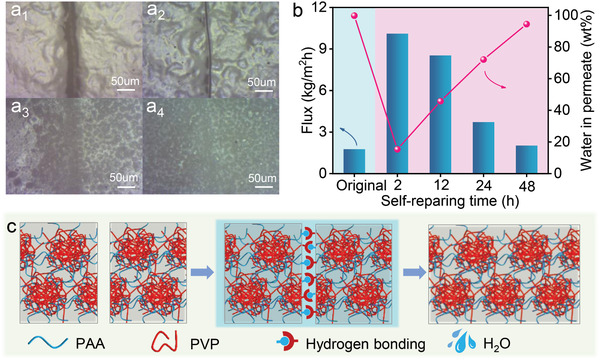
a) Microscopic images of a_1_) damaged HCPN membrane and after self‐repairing at water for different periods: a_2_) 2 h; a_3_) 12 h; and a_4_) 48 h. b) Separation performance recovery of the damaged HCPN membrane with different self‐repairing time at 45 ℃ in the water. c) The mechanism for the self‐repairing of damaged HCPN membrane with the assistance of water.

It is unavoidable to damage the membrane during manufacture, transportation, or separation process, shortening the lifespan of membranes and ultimately resulting in waste of materials and unsustainable development. The bulk PAA/PVP complex has been reported to have the self‐pairing ability by treating with water,^[^ ^42^ ^]^ and the prepared HCPN in our study also shows the self‐repairing capability, as shown in Figure S10, Supporting Information. Thus, taking full advantage of this peculiar ability, the self‐pairing ability of HCPN membrane was assessed by breaking the separation layer, as shown in **Figure** **4a**_1_. The damaged membrane was subsequently immersed into water for different period of time. Evidently, the scratched gap on the HCPN membrane becomes smaller after 2 h self‐repairing due to the reconstruction of hydrogen bonds between PAA and PVP chains (Figure 4a_2_) and is healed macroscopically after 12 h self‐repairing process (Figure 4a_3_). No obvious defects can be observed after 48 h self‐repairing in water, meaning the reusability of the damaged membranes. The self‐repairing mechanism of the membrane is portrayed in Figure 4c. It is well‐approved that the hydrogen bond force is reversible, which can reconnect after broken when the two chains are approaching with each other.^[^ ^52^, ^53^ ^]^ According to the principle, the polymer chains at damaged region of HCPN membrane could be recombined with the assistance of water which facilitates the polymer chains moving toward each other. The separation performance of self‐paired HCPN membrane was conducted with different self‐repairing periods. As expected, the flux markedly diminishes while the water content in permeate significantly enhances with prolonging the self‐repairing time. The performance of damaged HCPN membrane could fully recover to its original level after 48 h self‐repairing (Figure 4b), in line with the images in Figure 4a_4_. Therefore, the lifetime of HCPN membrane can be extended even with damage, promising for industry process.

In summary, a novel kind of supramolecular nanoparticle material, HCPN, was designed and synthesized, which was adopted for fabrication of the acid‐resistance and self‐repairing membrane. The size of HCPN could be efficiently and precisely tailored via controlling the complexation pH due to pH‐responsive characters of PAA, which thus tuned the performance of HCPN membranes. The optimized HCPN membrane was employed for dehydration of isopropanol, which presented a unique separation performance, i.e., the separation factor evidently boosts with pH reduction, and an enhancement of threefold was achieved with pH decreasing from 7 to 1. The membrane shows extremely strong acid‐resistance ability during over 100 h continuous running at pH = 1 condition. The PSI of the HCPN membrane could reach 1.1 × 10^7^ g m^−2^ h^−1^, an unprecedentedly high record reported. Notably, the damaged HCPN membrane can entirely restore its separation performance after self‐repairing for 48 h. Given the high separation performance, acid‐resistance feature, as well as self‐repairing ability of HCPN membrane, the proposed membrane materials open a new gate for development of robust membranes that work at harsh conditions with longevity, meeting the current sustainable development requirement.

## 3 Experimental Section

### Materials

Poly(acrylic acid) (PAA, *M*_w_ = 450 000) and poly(vinylpyrrolidone) (PVP, *M*_w_ = 40 000) were purchased from Sigma‐Aldrich. Hydrochloric acid (HCl) and sodium hydroxide (NaOH) were purchased from Beijing Chemical Factory. Isopropanol was obtained from Tianjin Damao Reagent Factory. All chemicals were used as received without further purification. Deionized water (DI water) with a resistance of 18 MΩ cm was used in all experiments. A polyacrylonitrile (PAN) ultrafiltration membrane with a molecular weight cutoff of 50 000 Da (PAN‐50) were purchased from Zhongke Rui Yang Membrane Technology Co., Ltd.

### Preparation of Hydrogen‐Bonding Complexed PAA/PVP Nanoparticles (HCPN)

The HCPN were prepared by tuning the pH of PAA and PVP mixed aqueous solution. First, PAA aqueous solution (4 mg mL^−1^, pH = 8) and PVP aqueous solution (6 mg mL^−1^, pH = 8) were homogeneously mixed together with 1:1 volume ratio, obtaining transparent solution due to the ionization of PAA chains hindering hydrogen bond formation. Next, the pH of the mixed solution was tuned by adding 1 m HCl drop by drop. Due to the pronation of PAA chains, PAA and PVP were gradually complexed through hydrogen bonds and the mixed solution became turbidity. The final pH of the mixed solution was controlled in the range of 3–5, ensuring the dispersion of HCPN in the water. And precipitation appeared when the pH was lower than 3.

### Preparation of HCPN Membrane

The HCPN dispersions was further diluted with DI water to 0.05 mg mL^−1^ and stored for future use. The PAN membrane was treated with oxygen plasma (80 W, 3 min) before use. The HCPN membrane was fabricated by directly deposited the above diluted HCPN dispersions onto the PAN membrane via vacuum filtration technique. The hydrogen bond complex membranes with different thickness were obtained by controlling the filtration volume.

### Pervaporation Experiment

The membrane was loaded into a homemade setup for pervaporation test with an effective area of 3.79 cm^2^. The upstream side of the membrane was in direct contact with the circulating solution at a constant temperature and the downstream side kept the pressure at about 100 Pa through a vacuum pump. The liquid was condensed with liquid nitrogen and collected into a cold trap and the components were analyzed by gas chromatography (GC‐14C, Shimadzu, Japan). The detector of the GC used is a kind of thermal conductivity detector and the column is the stainless‐steel packed column with stationary phase PoraparkQ, respectively. The GC calibration was accomplished by determination of different mass ratio of alcohol‐water mixed solution and drawing the standard curve. Permeation flux (*J*), separation factor (*α*), and PSI are calculated by the following formulas

(1) $$J=\frac{\Delta m}{A\times \Delta t}\;$$

(2) $$\alpha \;=\frac{P(H_{2}O)/P(\mathrm{IPA})}{F(H_{2}O)/F(\mathrm{IPA})}$$

(3) PSI=J×(α−1)

where Δ*m* is the weight of permeate collected during the Δ*t* time interval and *A* is the effective area of the membrane. *P*(H_2_O) and *P*(IPA) are the mass percentage of water and isopropanol at the permeation side and the *F*(H_2_O) and *F*(IPA) are the mass percentage of water and isopropanol at the feed side.

### Self‐Repairing Tests

For the free‐standing membrane, HCPN dispersions were sandwiched between two glass slides, which was pressed to form a flat membrane. The dried free‐standing HCPN membranes was cut into two halves with blades, which were put together, fixed, and immersed into water at 45 ℃ with different periods for self‐repairing. For HCPN membrane deposited on the substrate, the thin HCPN layer was scratched, and the whole membrane was then immersed into water at 45 ℃ with different periods before pervaporation tests.

### Characterization

An attenuated total reflectance FTIR spectrometer was used to characterize hydrogen bonding interactions (Vertex‐70, Bruker, Germany). The particle size of HCPN was measured by DLS (Z3000, Particle Sizing System, USA). The morphology of HCPN and the surface and cross‐section morphologies of the prepared membrane were studied by SEM (SU‐8020, Hitachi, Japan). Atomic force microscopy (AFM, SPI3800N, Seiko Instruments Inc., Japan) was used to measure the surface morphology of the membrane under swelling condition (feed environment, 10 wt% water/90 wt% isopropanol). Water contact angle analyzer (OCA 20, Data Physics, Germany) was used to measure the hydrophilicity of the membrane surface. An NMR analyzer (VTMR20‐010V‐I, Niumag Analytical Instruments, Inc., China) was used to analyze the interaction of HCPN with water in feed solutions with different pH. The tensile test was carried out on a universal tester with a tensile speed of 50 mm min^−1^ (34SC‐1, INSTRON, USA). Thermogravimetric analyzer was used to analyze the thermal stability under nitrogen. Each sample (≈10 mg) was heated from 25 to 800 ℃ at a rate of 10 ℃ min^−1^.

## Conflict of Interest

The authors declare no conflict of interest.

## Supporting information

Supporting Information

## Data Availability

Research data are not shared.

## References

[1] J. R. Werber , C. O. Osuji , M. Elimelech , Nat. Rev. Mater. 2016, 1, 16.

[2] L. Cao , X. Y. He , Z. Y. Jiang , X. Q. Li , Y. F. Li , Y. X. Ren , L. X. Yang , H. Wu , Chem. Soc. Rev. 2017, 46, 6725. 29022021 10.1039/c5cs00906e

[3] J. Y. Zhu , S. S. Yuan , J. Wang , Y. T. Zhang , M. M. Tian , B. Van der Bruggen , Prog. Polym. Sci. 2020, 110, 101308.

[4] W. S. Hung , S. Y. Ho , Y. H. Chiao , C. C. Chan , W. Y. Woon , M. J. Yin , C. Y. Chang , Y. M. O. Lee , Q. F. An , Chem. Mater. 2020, 32, 5750.

[5] T. Rodenas , I. Luz , G. Prieto , B. Seoane , H. Miro , A. Corma , F. Kapteijn , F. Xamena , J. Gascon , Nat. Mater. 2015, 14, 48. 25362353 10.1038/nmat4113 PMC4270742

[6] P. M. Budd , E. S. Elabas , B. S. Ghanem , S. Makhseed , N. B. McKeown , K. J. Msayib , C. E. Tattershall , D. Wang , Adv. Mater. 2004, 16, 456.

[7] D. S. Sholl , R. P. Lively , Nature 2016, 532, 435. 27121824 10.1038/532435a

[8] M. Carta , R. Malpass‐Evans , M. Croad , Y. Rogan , J. C. Jansen , P. Bernardo , F. Bazzarelli , N. B. McKeown , Science 2013, 339, 303. 23329042 10.1126/science.1228032

[9] C. Bar , N. Caglar , M. Uz , S. K. Mallapragada , S. A. Altinkaya , ACS Appl. Mater. Interfaces 2019, 11, 31367. 31424905 10.1021/acsami.9b10273

[10] S. Tang , Z. Dong , X. Zhu , Q. Zhao , J. Membr. Sci. 2019, 576, 59.

[11] X.‐S. Wang , Q.‐F. An , Q. Zhao , K.‐R. Lee , J.‐W. Qian , C.‐J. Gao , J. Membr. Sci. 2012, 415–416, 145.

[12] X. S. Cai , Y. T. Zhang , L. W. Yin , D. D. Ding , W. H. Jing , X. H. Gu , Chem. Eng. Sci. 2016, 153, 1.

[13] X.‐Q. Li , P.‐Y. Zheng , J.‐K. Wu , N.‐X. Wang , S.‐L. Ji , Z.‐h. Yu , Q.‐F. An , J. Membr. Sci. 2019, 573, 55.

[14] S. L. Yi , X. H. Ma , I. Pinnau , W. J. Koros , J. Mater. Chem. A 2015, 3, 22794.

[15] N. Jullok , S. Darvishmanesh , P. Luis , B. Van der Bruggen , Chem. Eng. J. 2011, 175, 306.

[16] J. Sun , C. Su , X. Zhang , J. Li , W. B. Zhang , N. Zhao , J. Xu , S. Yang , J. Colloid Interface Sci. 2018, 513, 470. 29175741 10.1016/j.jcis.2017.10.021

[17] X.‐S. Wang , Q.‐F. An , F.‐Y. Zhao , Q. Zhao , K.‐R. Lee , J.‐W. Qian , C.‐J. Gao , Cellulose 2014, 21, 3597.

[18] B. Bolto , T. Tran , M. Hoang , Z. L. Xie , Prog. Polym. Sci. 2009, 34, 969.

[19] K. C. Khulbe , C. Feng , T. Matsuura , J. Appl. Polym. Sci. 2010, 115, 855.

[20] Q. Zhao , M. J. Yin , A. P. Zhang , S. Prescher , M. Antonietti , J. Y. Yuan , J. Am. Chem. Soc. 2013, 135, 5549. 23544436 10.1021/ja402100r

[21] Q. Zhao , D. W. Lee , B. K. Ahn , S. Seo , Y. Kaufman , J. N. Israelachvili , J. H. Waite , Nat. Mater. 2016, 15, 407. 26779881 10.1038/nmat4539 PMC4939084

[22] Q. Zhao , J. Heyda , J. Dzubiella , K. Tauber , J. W. Dunlop , J. Yuan , Adv. Mater. 2015, 27, 2913. 25828569 10.1002/adma.201500533

[23] X. S. Wang , Y. L. Ji , P. Y. Zheng , Q. F. An , Q. Zhao , K. R. Lee , J. W. Qian , C. J. Gao , J. Mater. Chem. A 2015, 3, 7296.

[24] X. S. Wang , Q. F. An , Q. Zhao , K. R. Lee , J. W. Qian , C. J. Gao , J. Membr. Sci. 2013, 435, 71.

[25] M. Khatib , O. Zohar , H. Haick , Adv. Mater. 2021, 33, 2004190. 10.1002/adma.202004190 33533124

[26] L. Zhang , Z. Liu , X. Wu , Q. Guan , S. Chen , L. Sun , Y. Guo , S. Wang , J. Song , E. M. Jeffries , C. He , F. L. Qing , X. Bao , Z. You , Adv. Mater. 2019, 31, e1901402. 10.1002/adma.201901402 30977571

[27] T. P. Huynh , P. Sonar , H. Haick , Adv. Mater. 2017, 29, 1604973. 10.1002/adma.201604973 28229499

[28] W. Li , B. Dong , Z. Yang , J. Xu , Q. Chen , H. Li , F. Xing , Z. Jiang , Adv. Mater. 2018, 30, e1705679. 10.1002/adma.201705679 29577476

[29] N. Zheng , Y. Xu , Q. Zhao , T. Xie , Chem. Rev. 2021, 121, 1716. 33393759 10.1021/acs.chemrev.0c00938

[30] M. W. Urban , D. Davydovich , Y. Yang , T. Demir , Y. Z. Zhang , L. Casabianca , Science 2018, 362, 220. 30309952 10.1126/science.aat2975

[31] F. Luo , T. L. Sun , T. Nakajima , T. Kurokawa , Y. Zhao , K. Sato , A. B. Ihsan , X. Li , H. Guo , J. P. Gong , Adv. Mater. 2015, 27, 2722. 25809867 10.1002/adma.201500140

[32] D. Davydovich , M. W. Urban , Nat. Commun. 2020, 11, 7. 10.1038/s41467-020-19405-5 PMC7665198 33184268

[33] P. A. Korevaar , S. J. George , A. J. Markvoort , M. M. J. Smulders , P. A. J. Hilbers , A. Schenning , T. F. A. De Greef , E. W. Meijer , Nature 2012, 481, 492. 22258506 10.1038/nature10720

[34] M. Nakahata , Y. Takashima , H. Yamaguchi , A. Harada , Nat. Commun. 2011, 2, 6. 10.1038/ncomms1521 PMC3207205 22027591

[35] M. Burnworth , L. M. Tang , J. R. Kumpfer , A. J. Duncan , F. L. Beyer , G. L. Fiore , S. J. Rowan , C. Weder , Nature 2011, 472, 334. 21512571 10.1038/nature09963

[36] Y. Chen , A. M. Kushner , G. A. Williams , Z. Guan , Nat. Chem. 2012, 4, 467. 22614381 10.1038/nchem.1314

[37] G. Yan , Y. Feng , H. Wang , Y. Sun , X. Tang , W. Hong , X. Zeng , L. Lin , Commun. Mater. 2020, 1, 41.

[38] P.‐F. Cao , B. Li , T. Hong , J. Townsend , Z. Qiang , K. Xing , K. D. Vogiatzis , Y. Wang , J. W. Mays , A. P. Sokolov , T. Saito , Adv. Funct. Mater. 2018, 28, 1800741.

[39] C.‐H. Huang , Y.‐L. Liu , RSC Adv. 2017, 7, 38360.

[40] M. J. Yin , M. Yao , S. R. Gao , A. P. Zhang , H. Y. Tam , P. K. A. Wai , Adv. Mater. 2016, 28, 1394. 26643765 10.1002/adma.201504021

[41] Z. G. Yin , M. J. Yin , Z. Y. Liu , Y. X. Zhang , A. P. Zhang , Q. D. Zheng , Adv. Sci. 2018, 5, 11.

[42] N. An , X. Wang , Y. Li , L. Zhang , Z. Lu , J. Sun , Adv. Mater. 2019, 31, 1904882. 10.1002/adma.201904882 31456254

[43] A. Henke , S. Kadłubowski , M. Wolszczak , P. Ulański , V. Boyko , T. Schmidt , K.‐F. Arndt , J. M. Rosiak , Macromol. Chem. Phys. 2011, 212, 2529.

[44] T. S. He , X. D. Yu , T. J. Bai , X. Y. Li , Y. R. Fu , K. D. Cai , Ionics 2020, 26, 4103.

[45] M. J. Yin , Z. G. Yin , Y. X. Zhang , Q. D. Zheng , A. P. Zhang , Nano Energy 2019, 58, 96.

[46] J.‐K. Wu , N.‐X. Wang , W.‐S. Hung , Q. Zhao , K.‐R. Lee , Q.‐F. An , J. Mater. Chem. A 2018, 6, 22925.

[47] J. K. Wu , M. J. Yin , W. Han , N. X. Wang , Q. F. An , J. Mater. Sci. 2020, 55, 12607.

[48] W. H. Zhang , M. J. Yin , Q. Zhao , C. G. Jin , N. X. Wang , S. L. Ji , C. L. Ritt , M. Elimelech , Q. F. An , Nat. Nanotechnol. 2021, 16, 337. 33479540 10.1038/s41565-020-00833-9

[49] M.‐J. Yin , Z.‐R. Li , T.‐R. Lv , K.‐T. Yong , Q.‐F. An , Sens. Actuators, B 2021, 339, 129887.

[50] C. Cheng , P. Li , K. Shen , T. Zhang , X. Cao , B. Wang , X. Wang , B. S. Hsiao , J. Membr. Sci. 2018, 553, 70.

[51] J. Li , X. Si , X. Li , N. Wang , Q. An , S. Ji , Sep. Purif. Technol. 2018, 192, 205.

[52] P. Song , H. Wang , Adv. Mater. 2020, 32, 1901244. 10.1002/adma.201901244 31215093

[53] R. Tamate , K. Hashimoto , T. Horii , M. Hirasawa , X. Li , M. Shibayama , M. Watanabe , Adv. Mater. 2018, 30, 1802792. 10.1002/adma.201802792 30066342

