# Supplementary material for: Acid‐Resistance and Self‐Repairing Supramolecular Nanoparticle Membranes via Hydrogen‐Bonding for Sustainable Molecules Separation
Source: Adv Sci (Weinh). 2021 Oct 19;8(23):2102594. doi: 10.1002/advs.202102594 (PMC8655207; doi:10.1002/advs.202102594)
Supplement: Supplementary file 1 — Supporting Information [file ADVS-8-2102594-s001.pdf]

© 2021 Wiley-VCH GmbH

ADVANCED SCIENCE Open Access

Supporting Information

for *Adv. Sci.*, DOI: 10.1002/advs.202102594

Acid-resistance and self-repairing supramolecular nanoparticle membranes via hydrogen-bonding for sustainable molecules separation

Wang Han[a], *Ming-Jie Yin*[a,*], *Wen-Hai Zhang*[a], *Zhi-Jie Liu*[a], *Ken Tye Yong*[c,d], *and Quan-Fu An*[a,*]

Acid-resistance and self-repairing supramolecular nanoparticle membranes via hydrogen-bonding for sustainable molecules separation

Wang Han[a], *Ming-Jie Yin*[a,]*, *Wen-Hai Zhang*[a], *Zhi-Jie Liu*[a], *Ken Tye Yong*[c,d], *and Quan-Fu An*[a,]*

Mr. W. Han, Dr. M.J Yin, Mr. W.H. Zhang, Mr. Z.J. Liu, Prof. Q.F. An

Beijing Key Laboratory for Green Catalysis and Separation, Department of Environmental and Chemical Engineering, Beijing University of Technology, Beijing 100124, China

E-mail: yinmj@bjut.edu.cn; anqf@bjut.edu.cn

Prof. K.T. Yong

The University of Sydney Nano Institute, The University of Sydney, Sydney, New South Wales 2006, Australia

School of Biomedical Engineering, The University of Sydney, Sydney, New South Wales 2006, Australia

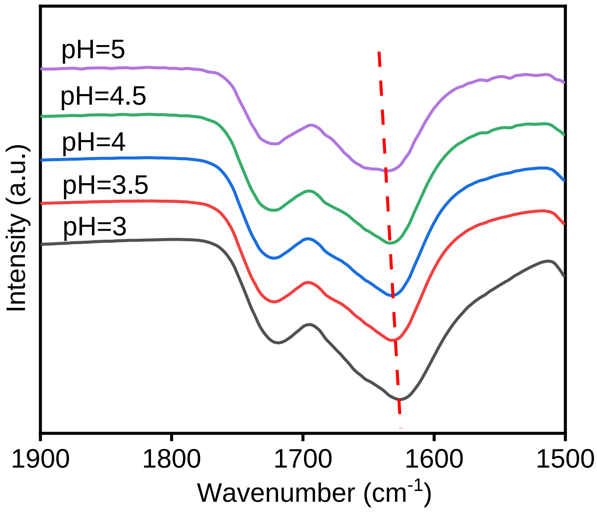

Figure S1. FTIR spectra variation of HPCN prepared at different pH conditions.

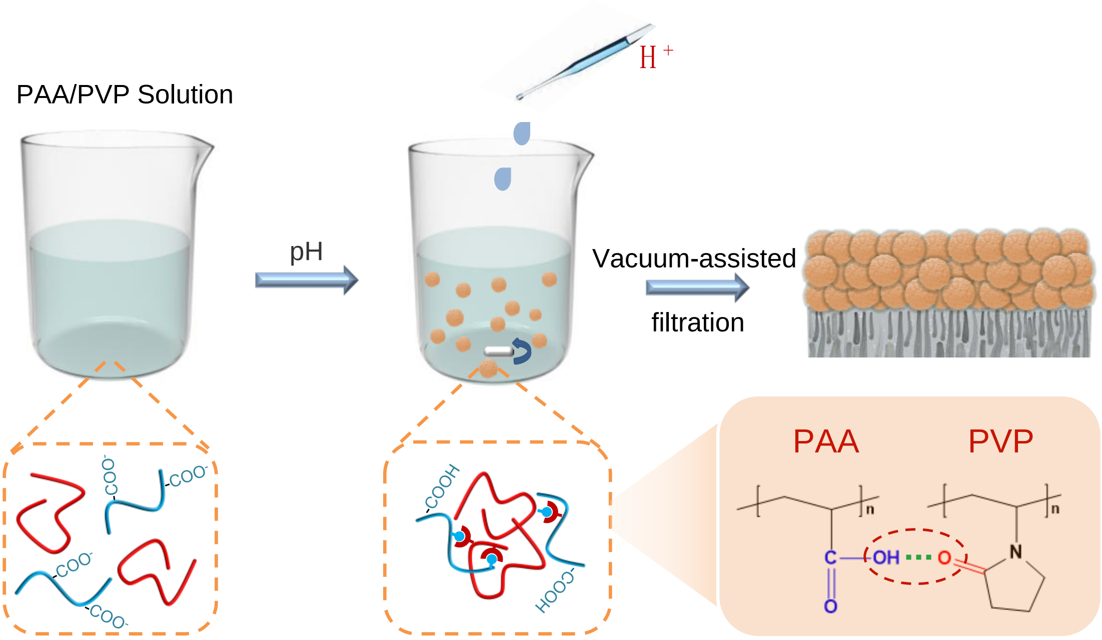

Figure S2. Scheme of the HCPN membrane process via vacuum-assisted filtration technique.

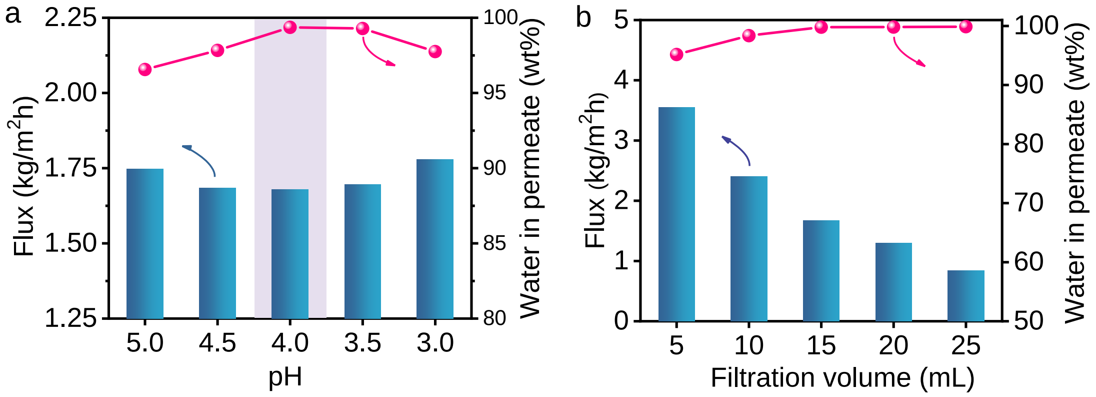

Figure S3. Performance of HCPN membrane in dehydration of 10% water/isopropanol mixtures at 50°C with different preparation conditions: (a) HCPN complexed at different pH and (b) filtration volume of dispersed HCPN solution.

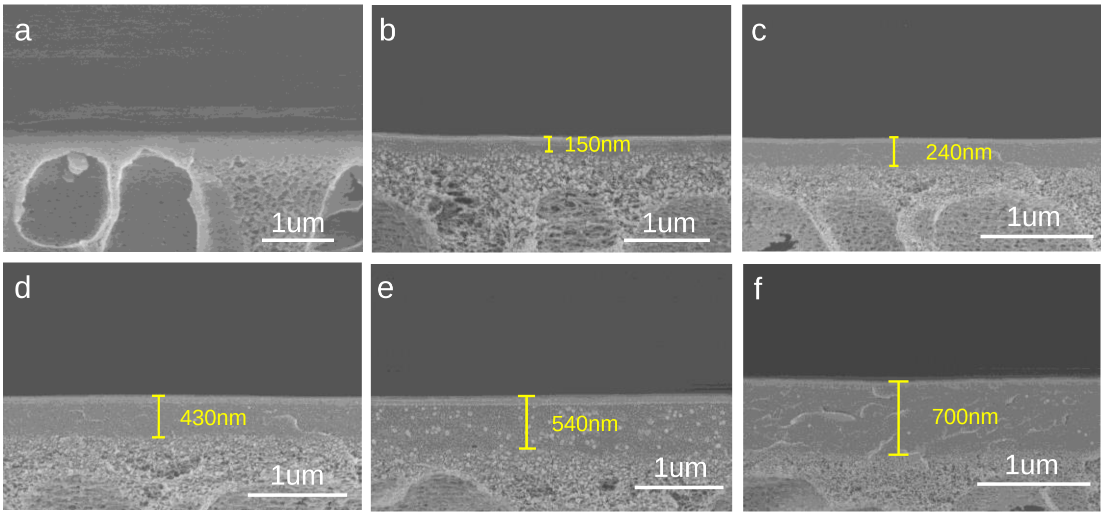

Figure S4. The cross-section view of the deposited HCPN membrane on PAN substrate with different filtration volume: (a) 0 mL; (b) 5 mL; (c) 10 mL; (d) 15 mL; (d) 20 mL; and (e) 25 mL.

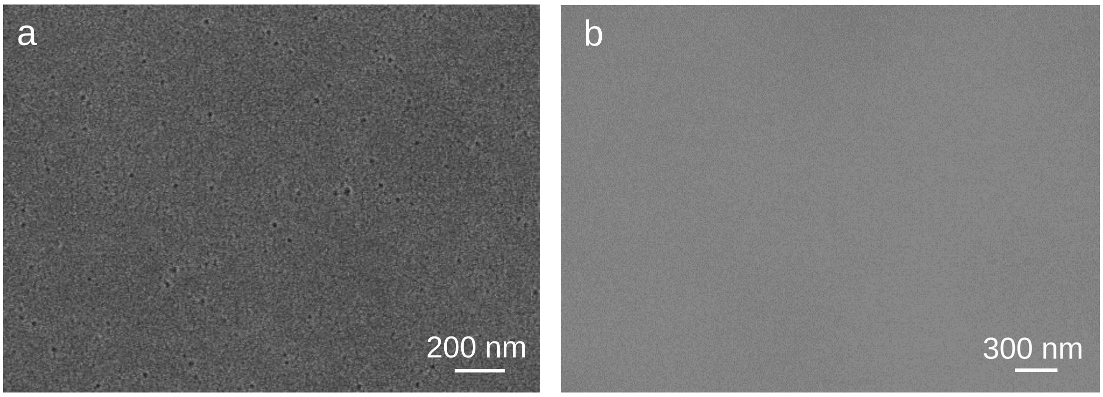

Figure S5. The surface morphology of HCPN membrane before (a) and after (b)

deposition of HCPN.

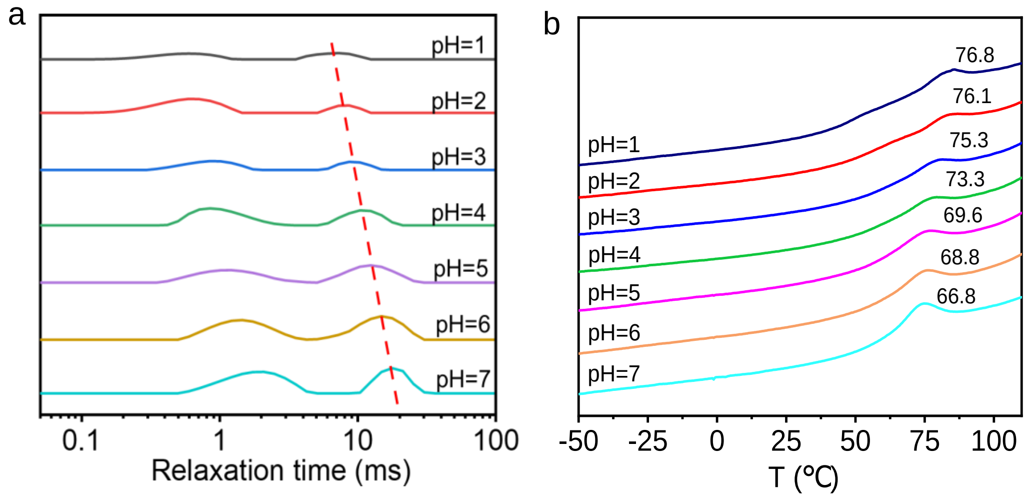

Figure S6. (a) T_2 relaxation time evolution of HCPN membrane at feed solution with different pH. (b) DSC curves of HCPN membranes treated with different pH.

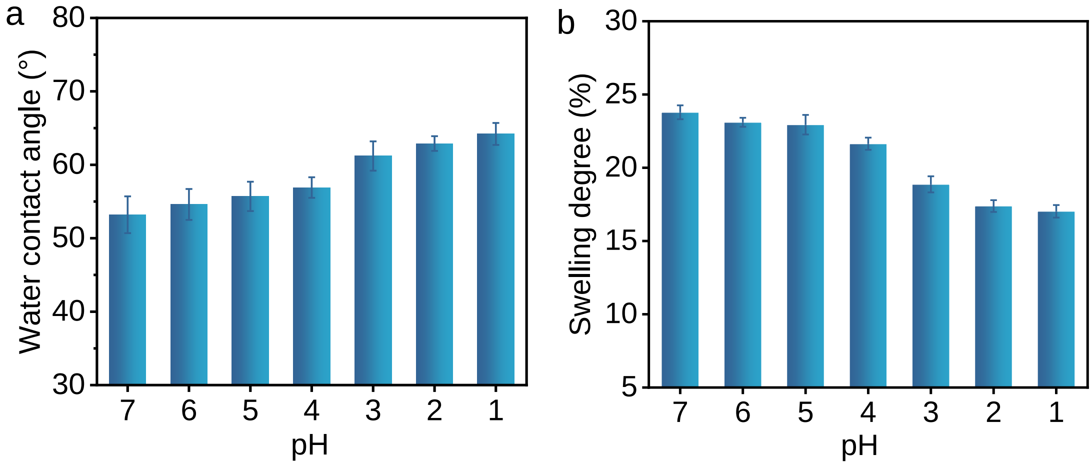

Figure S7. pH dependence of the water contact angle (a) and swelling degree (b) of HCPN membrane.

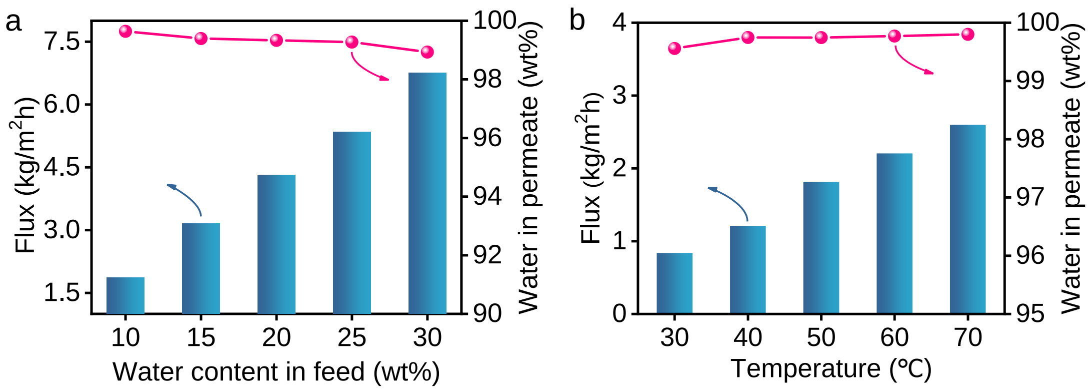

Figure S8. The influence of water content (a) and measured temperature on the separation performance of HCPN membrane.

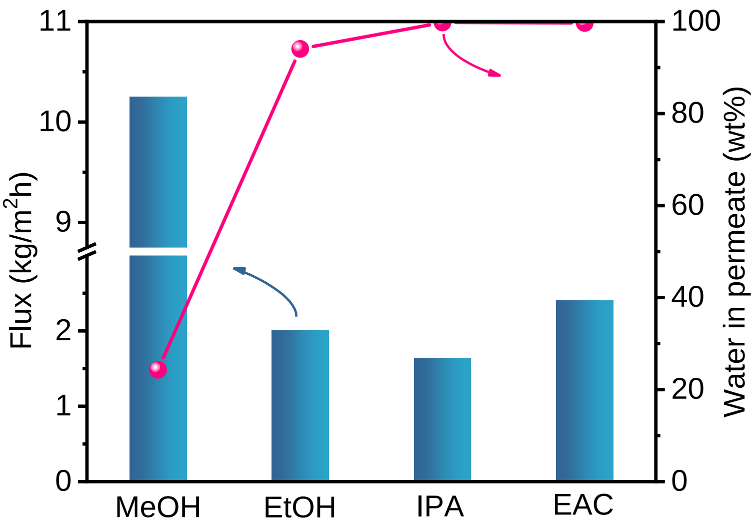

Figure S9. The separation performance of HCPN membrane in dehydration of different organic solvents mixed with 10 wt% water.

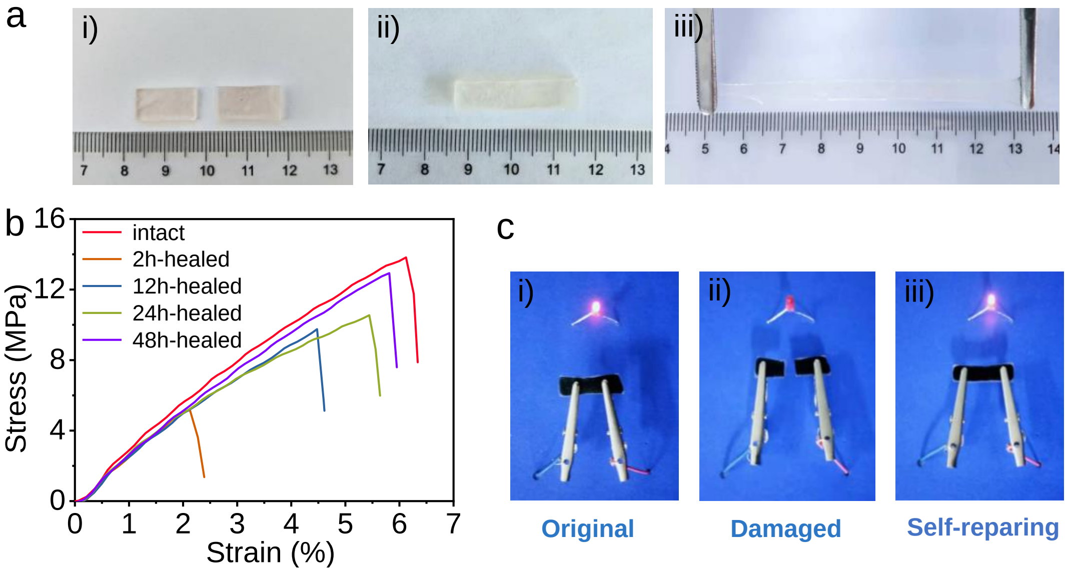

Figure S10. The self-repairing ability of HCPN: (a) pictures of fractured HCPN before and after self-repairing; (b) stress–strain curves of the intact and 2, 12, 24, and 48 h self-repaired HCPN in water; (c) PEDOT: PSS doped HCPN used as conducting wire, which can be self-repaired in water after cut into two parts.

Table S1 Separation performance comparison of different polymer pervaporation membrane in dehydration of alcohols at acidic condition.

Membranes	pH	Feed composition	T (°C)	Flux (kg/m^{2}h)	Water content in permeate (%)	Refs.
PILC	1	90% IPA	70	1.1	991	[1]
SPECs	1.2	90% IPA	40	0.5	1491	[2]
S-CMC/CS	2	90% EtOH	50	0.6~0.7	205	[3]
PEC/NaA	2	90% EtOH	40	0.52	591	[4]
PEC/NaA	3	90% EtOH	40	0.85	4491	[4]
SA/PEI	1	90% EtOH	60	1.7	991	[5]
Zeolite PHI	3.5	90% EtOH	80	0.1	991	[6]
HCPNM	1	90% EtOH	50	2.01	163	this study
HCPNM	1	90% IPA	50	1.65	4491	this study

Reference

[1] S. Tang, Z. Dong, X. Zhu, Q. Zhao, J. Membr. Sci. 2019, 576, 59.

[2] X.-S. Wang, Q.-F. An, Q. Zhao, K.-R. Lee, J.-W. Qian, C.-J. Gao, J. Membr. Sci. 2012, 415-416, 145.

[3] X.-S. Wang, Q.-F. An, F.-Y. Zhao, Q. Zhao, K.-R. Lee, J.-W. Qian, C.-J. Gao, Cellulose. 2014, 21, 3597.

[4] X.-Q. Li, P.-Y. Zheng, J.-K. Wu, N.-X. Wang, S.-L. Ji, Z.-h. Yu, Q.-F. An, J. Membr. Sci. 2019, 573, 55.

[5] J. Li, X. Si, X. Li, N. Wang, Q. An, S. Ji, Sep. Purif. Technol. 2018, 192, 205.

[6] Y. Kiyozumi, Y. Nemoto, T. Nishide, T. Nagase, Y. Hasegawa, F. Mizukami, Microporous Mesoporous Mater. 2008, 116, 485.
